# Save your host, save yourself? Caste‐ratio adjustment in a parasite with division of labor and snail host survival following shell damage

**DOI:** 10.1002/ece3.3782

**Published:** 2018-01-03

**Authors:** Colin MacLeod, Robert Poulin, Clément Lagrue

**Affiliations:** ^1^ Department of Zoology University of British Columbia Vancouver BC Canada; ^2^ Department of Zoology University of Otago Dunedin New Zealand

**Keywords:** caste‐ratio adjustment, division of labor, host survival, *Philophthalmu*s sp., shell damage, shell repair, trematode parasites, *Zeacumantus subcarinatus*

## Abstract

Shell damage and parasitic infections are frequent in gastropods, influencing key snail host life‐history traits such as survival, growth, and reproduction. However, their interactions and potential effects on hosts and parasites have never been tested. Host–parasite interactions are particularly interesting in the context of the recently discovered division of labor in trematodes infecting marine snails. Some species have colonies consisting of two different castes present at varying ratios; reproductive members and nonreproductive soldiers specialized in defending the colony. We assessed snail host survival, growth, and shell regeneration in interaction with infections by two trematode species, *Philophthalmu*s sp. and *Maritrema novaezealandense*, following damage to the shell in the New Zealand mud snail *Zeacumantus subcarinatus*. We concomitantly assessed caste‐ratio adjustment between nonreproductive soldiers and reproductive members in colonies of the trematode *Philophthalmu*s sp. in response to interspecific competition and shell damage to its snail host. Shell damage, but not parasitic infection, significantly increased snail mortality, likely due to secondary infections by pathogens. However, trematode infection and shell damage did not negatively affect shell regeneration or growth in *Z. subcarinatus*; infected snails actually produced more new shell than their uninfected counterparts. Both interspecific competition and shell damage to the snail host induced caste‐ratio adjustment in *Philophthalmu*s sp. colonies. The proportion of nonreproductive soldiers increased in response to interspecific competition and host shell damage, likely to defend the parasite colony and potentially the snail host against increasing threats. These results indicate that secondary infections by pathogens following shell damage to snails both significantly increased snail mortality and induced caste‐ratio adjustments in parasites. This is the first evidence that parasites with a division of labor may be able to produce nonreproductive soldiers according to environmental factors other than interspecific competition with other parasites.

## 1 INTRODUCTION

The evolutionary success of molluscs in general and gastropods in particular relies to a large extent on a specific organ, the shell, and on the way this mineralized exoskeleton is produced (Fleury et al., 2008; Marin, Le Roy, & Marie, 2012). As the gastropod increases in size, the shell grows larger around the aperture's edge and continuously thicker on the inside through secretions from the mantle to accommodate and protect the increasing volume of the organism's soft tissues and compensate for shell erosion (Day, Branch, & Viljoen, 2000; Simkiss & Wilbur, 1989). One aspect that makes this structure so successful comes from the ability of gastropods to repair shell damage and overcome external aggressions such as accidental shell cracks or incomplete predation attempts (Fleury et al., 2008; Watabe, 1983). Snails repair their shells following damage from external factors through specific secretion and muscular activities of the calcifying epithelium (Andrews, 1934, 1935; Cadée, 1999; Cadée, Walker, & Flessa, 1997; Fleury et al., 2008). However, the energetic demands of shell repair can reduce energy available for reproduction and growth; past studies have shown that nonlethal injuries may result in decreased growth and survival (Geller, 1990). Nonlethal shell damage from predator attacks or erosion is particularly common in marine gastropods; up to ~20% in the intertidal snail *Nucella emarginata* and between 15% and 75% in *Patella* and *Nacella* spp. limpets for examples (Cadée, 1999; Day et al., 2000; Geller, 1983). However, potential long‐term effects on snail survival and shell growth are often unknown (Vermeij, Schindel, & Zipser, 1981).

Snails are often infected by helminth parasites (Bayne, 1983). The gastropod shell provides little defense against helminth infections, although a few striking examples have shown that the shell can successfully kill nematode parasites (Rae, 2017; Williams & Rae, 2016). For example, there are probably tens of thousands of species of trematodes (Trematoda: Digenea), the vast majority of which utilizing gastropods as obligatory hosts in their life cycles (van der Knaap & Loker, 1990). Trematodes are particularly common in marine ecosystems and infect a wide variety of snail species in which they form clonal colonies (Galaktionov & Dobrovolskij, 2003; Miura, 2012; Mouritsen & Halvorsen, 2015). Trematode colonies can make up to 40% of the mass of an infected snail and infections by trematode parasites can significantly affect snail survival, growth, and fecundity as well as shell strength. Indeed, trematode infections alter the calcifying ability of marine gastropods, sometimes in species‐specific ways (Hechinger, Lafferty, Mancini, Warner, & Kuris, 2009; MacLeod & Poulin, 2015). Alteration of shell strength by parasites can in turn increase predation risk and nonlethal shell damage occurrence in marine snails (Kamiya & Poulin, 2012). Overall, both parasitic infections and nonlethal shell damage can influence snail survival and shell growth, strength, and regeneration (i.e., repair of damage). However, the individual and combined effects of parasites and shell damage on snail survival, shell repair, and growth have never been documented before in the same snail host‐trematode parasite system.

Our study system consists of the intertidal snail *Zeacumantus subcarinatus* (Batillariidae) and its two most common trematode parasites, *Maritrema novaezealandense* (Microphallidae) and *Philophthalmu*s sp. (Philophthalmidae; Martorelli, Fredensborg, Mouritsen, & Poulin, 2004; Martorelli, Fredensborg, Leung, & Poulin, 2008; Presswell, Blasco‐Costa, & Kostadinova, 2014). This snail is an abundant grazer of microalgae in many intertidal ecosystems throughout New Zealand and host to many species of parasites, with up to 80% of individuals infected in some localities (Fredensborg, Mouritsen, & Poulin, 2005; Leung et al., 2009). A large proportion of these snails also show evidence of nonlethal shell damage covered with regenerated shell; over 90% of adult snails display signs of continuous physical erosion and subsequent repair at the tip of the shell and around 5% carry repaired, traumatic shell damage from nonlethal predator attacks on one of the three largest shell whorls (Lagrue, personal observations). Trematodes use *Z. subcarinatus* as first intermediate host, within which they undergo repeated asexual multiplication, resulting in colonies of clonal stages known as parthenitae (Hechinger, Wood, & Kuris, 2011; Lafferty & Kuris, 2009). The main role of these parthenitae is to produce free‐living larvae that must leave the snail host to encyst in or on second intermediate hosts and continue the life cycle of the parasites (Martorelli et al., 2004, 2008). Trematodes occupy the place where the gonads are located in uninfected snails; infection by the two trematode species used in this study causes complete castration of the host (Fredensborg et al., 2005). *Maritrema novaezealandense* develops into sporocysts inside the snail while *Philophthalmu*s sp. (Philophthalmidae) develops into rediae (Martorelli et al., 2004, 2008). This is a key difference as rediae possess mouthparts and a digestive tract that enable them to actively feed on snail tissues and attack competitors; sporocysts do not possess these anatomical features (Galaktionov & Dobrovolskij, 2003; Nielsen, Johansen, & Mouritsen, 2014). In fact, rediae can feed on sporocysts of competing parasite species (Lim & Heyneman, 1972; Sousa, 1993). Furthermore, *Philophthalmu*s sp. produces two types of rediae that are morphologically and functionally distinct; in addition to the typical larvae‐producing rediae, this species also has smaller rediae with an elongated gut that do not produce free‐living larvae (Leung & Poulin, 2011; Martorelli et al., 2008). The nonreproductive soldiers are very active, using locomotor appendages to move around within the host. They have relatively larger mouthparts, readily attack members of foreign colonies, and are particularly common at invasion fronts within the host's body (Kamiya, O'Dwyer, Nuy, & Poulin, 2013; Leung & Poulin, 2011; Newey & Keller, 2010). The division of labor observed in *Philophthalmu*s sp. and other trematode species is similar to that characterizing other social systems with a soldier caste and likely benefits the colony's overall fitness (Hechinger et al., 2011; Lloyd & Poulin, 2012).

In most organisms with division of labor, castes are morphologically specialized for particular tasks within the colony (Oster & Wilson, 1978). Nonreproductive soldier rediae in trematode colonies are often assumed to be specialized for antagonistic interspecific interactions against other parasites attempting to infect the same snail host (Kamiya et al., 2013; Mouritsen & Halvorsen, 2015; Nielsen et al., 2014). This is consistent with the fact that interspecific competition for resources and space represents a potentially strong selection pressure for trematodes infecting snail hosts (Kuris & Lafferty, 1994; Mouritsen & Andersen, 2017; Poulin, 2001). However, even when not competing with other parasites, colonies of trematodes with a division of labor retain nonreproductive forms, albeit at different ratios (Leung & Poulin, 2011). In *Philophthalmu*s sp., while there is a clear benefit to maintaining the nonreproductive castes in the presence of a competitor, soldier morphs still provide a fitness benefit to the colony, in terms of larval output, in the absence of competition (Kamiya & Poulin, 2013; Lloyd & Poulin, 2012, 2014a). It is thus possible that these nonreproductive morphs fulfill additional roles in the colony. For example, parasite colonies as well as their molluscan hosts are likely to face repeated bacterial or fungal infections (Bayne, 1983; Ducklow, Boyle, Maugel, Strong, & Mitchell, 1979; van der Knaap & Loker, 1990; Morley, 2010); soldier rediae might play a “cleaning” role in the defense against such microorganisms, as documented in social insects (Lloyd & Poulin, 2012, 2014a; Mouritsen & Andersen, 2017; Turnbull et al., 2012). Since trematode infection can suppress host immune defenses, the risk of secondary infection by pathogens should be increased (Bayne, 1983; Iakovleva, Shaposhnikova, & Gorbushin, 2006; Loker & Adema, 1995; Walker, 1979, 2006). Ultimately, soldier rediae may defend not only their colony, but also indirectly the host within which the trematode colony resides and without which it is doomed (Hechinger et al., 2011). As such, soldier rediae are comparable to giant macrophages protecting the host against secondary microbial infections and working hard to keep it alive.

Here, we tested the potential role of these soldier rediae in protecting the parasite colony, alongside its host, against increased secondary infection rates following shell damage to the snail; shell damage being a portal to microbial and fungal infections in gastropods (Andrews, 1935). The theory of optimal caste‐ratio predicts that the ratio of caste members (soldier to reproductive rediae) responds to environmental variability (Lloyd & Poulin, 2013; Oster & Wilson, 1978). We thus predicted that the ratio of soldier morphs in *Philophthalmu*s sp. colonies should increase following shell damage to their snail hosts compared to colonies in snails with no shell damage. Such caste‐ratio adjustment may in turn increase *Philophthalmu*s‐infected snail survival compared to uninfected individuals following shell damage. Here, division of labor should respond to potential increase in secondary infection risks (Lloyd & Poulin, 2014a). In contrast, snails infected with the competitor, *M*. *novaezealandense*, may display survival rates comparable or lower to their uninfected counterparts following shell damage as their sporocysts cannot provide any physical defense against secondary pathogens and may actually render the host more vulnerable to pathogens if trematode infection reduces snail immune defenses (Iakovleva et al., 2006). We also tested for the potential added environmental pressure of shell damage on caste‐ratio in *Philophthalmu*s sp. colonies competing with *M*. *novaezealandense* in snails carrying co‐infections. Here, we predict that caste‐ratio may either become further biased in favor of soldiers following shell damage or remain stable if already at its maximum due to interspecific competition between the co‐infecting trematode species.

## 2 MATERIALS AND METHODS

### 2.1 Field collection, snail screening, and parasite identification

Around 10,000 New Zealand mud snails (*Zeacumantus subcarinatus*) were collected in Lower Portobello Bay (Otago Harbour, South Island, New Zealand; 45°52′S, 170°42′E) in October‐November 2015. Snails were screened for infection by *Philophthalmus* sp., *Maritrema novaezealandense* or both parasite species together (double infection) by incubating individuals overnight at 26°C and under constant light, conditions known to trigger the emergence of the parasites' free‐living larvae. During incubation, snails were kept individually in wells of 12‐well culture plates filled with natural seawater (Lloyd & Poulin, 2011; MacLeod & Poulin, 2015). Parasite species were identified by inspecting each well of the culture plates under a dissecting microscope and comparing larvae found with larval morphology from published descriptions of parasite species (Martorelli et al., 2008). Snails identified as infected (i.e., shedding parasite larvae) were separated by infection status (*Philophthalmus* sp. [~200 individuals], *M. novaezealandense* [~2500] or double infection [~200]), maintained at room temperature (~16°C) for 2 weeks before being screened a second time, and then a third time 2 weeks later, to confirm their infection status. Individuals showing signs of previous shell damage other than the ordinary shell erosion of the shell tip were discarded to eliminate potential confounding effects of previous traumatic injuries. A total of 99 snails per infection status were haphazardly selected and used in the following experiment. A subsample of uninfected snails (~1000) were also kept and screened the same way as infected individuals and an equivalent number (99) was haphazardly selected to provide an uninfected control group. All snails selected for the experiment were then marked with individual identification labels (numbered and colored plastic tags [Queen Marking Kit, The Bee Works©, Orillia, ON, Canada] fixed with cyanoacrylate glue; Lloyd & Poulin, 2012). Snails of different infection status were collectively identified through color‐coded tags (white for uninfected snails, blue for *M*. *novaezealandense*, yellow for *Philophthalmus* sp. and orange for double infections) and individually through a unique alphanumeric number (from 1 to 99 in each color code). Individually tagged snails were measured with calipers (total shell length to 0.01 mm) and then maintained together in a large fifty liter tank at room temperature (~16°C) in aerated seawater and fed sea lettuce (*Ulva* spp.) ad libitum until the start of the experiment.

### 2.2 Shell manipulation treatment, shell growth, and repair

**Figure 1:**
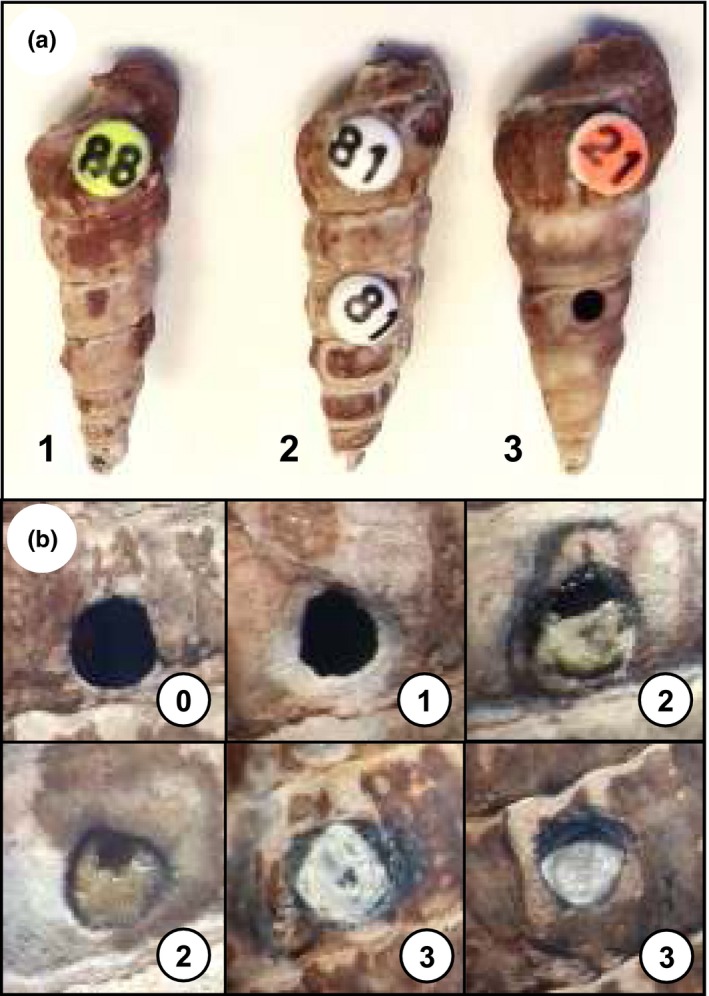
(a) Illustration of snail tagging and shell treatment: 1 *Philophthalmus* sp.‐infected snail in the undrilled (control) treatment, 2 uninfected individual in the drilled and patched treatment with the second alphanumeric tag used to patch the hole in the shell, and 3 snail infected by both parasite species (double infection [*Philophthalmus* sp. and *Maritrema novaezealandense*]) in the drilled treatment; the hole drilled in the shell was left exposed. (b) Illustration of the four degrees of shell repair from 0 no apparent repair and no production of new shell, 1 new shell produced but less than fifty percent of the hole covered, 2 over fifty percent of shell damage covered, and 3 repair complete and hole entirely covered

To standardize shell damage among all snails, we used a high‐speed drill (30,000 rpm; Dremel^®^ 3000 Rotary Tool) mounted on a vertical press (Dremel^®^ 3‐in‐1 Workstation) and a fine drill bit (diameter = 1 mm). During shell drilling, snails were maintained in the same position with their operculum facing down and a hole (shell damage) was made in the middle of the third whorl of the shell with the drill bit perpendicular to the shell surface. The drill bit was carefully pushed through the shell to a maximum depth of one millimeter to go through the shell while avoiding any damage to snail tissues underneath (Fleury et al., 2008). Prior to shell treatment, all snails were removed from the holding tank and dried under a fan; *Z. subcarinatus* are intertidal snails that can withstand prolonged periods out of the water. A third of the snails from each infection group were haphazardly assigned to the undrilled treatment and their shell left intact (Figure 1a). However, apart from shell damage, they were treated the same way as the others to eliminate potential effects of handling during shell treatment on snail mortality and growth. The remaining two‐thirds of the snails had a hole drilled into their shell as described above. After drilling, snails were cleaned of shell dust produced by the high speed drill. Half of the snails with shell damage (a third of total snail numbers) were left with the hole in the shell exposed and assigned to the drilled treatment (Figure 1a). Shell damage was patched in the remaining drilled snails to prevent direct contact with water and potential secondary microbial infection (Andrews, 1935). Plastic tags (Queen Marking Kit, The Bee Works©, Orillia, ON, Canada) were fastened with cyanoacrylate glue over the hole in the shell; tags used to cover shell damage matched the unique identification label of each snail. The glue was applied on the outer surface of the shell around the hole drilled. As a result, it did not get in contact with snail tissues and did not seem to elicit an immune response from the snail or impact shell regeneration. These snails were assigned to the patched treatment (Figure 1a).

To document shell growth during the experiment and assess potential effects of parasites and shell damage on shell production, all snails were photographed (Olympus camera, DP25) in a standardized position and at a fixed magnification at the beginning of the experiment and after 12 weeks for surviving individuals (MacLeod & Poulin, 2015). At the beginning of the experiment, and just after shell treatment, all snails were soaked for 24 hr in a saltwater solution of Calcein^™^ (120 mg/L). Calcein^™^ is a soluble fluorochrome which is incorporated into growing calcified structures and produces a fluorescent band which can be treated as a baseline for subsequent shell growth measurement (Riascos, Guzman, Laudien, Heilmayer, & Oliva, 2007). After 12 weeks, surviving snails were photographed again, in the same standardized position and under UV light (Leica camera, DFC350). ImageJ software (U.S. National Institutes of Health, Bethesda, MD, USA) was then used to measure the average distance in micrometers between the baseline fluorescent band and the new growing edge of the shell (shell growth) from the UV images (MacLeod & Poulin, 2015).

Repair of shell damage was also assessed for snails that were subjected to shell drilling and estimated as stages of shell repair. When shell damage occurs far from the aperture, new material secreted by the snail mantle to bridge the gap in the shell is colorless and usually produced under the old shell, and thus easy to distinguish from original shell material (Andrews, 1934, 1935; Fleury et al., 2008). Four degrees of shell repair were arbitrarily defined as follows: 0 when no repair and no production of new shell was observed, 1 when new shell had been produced but covered less than fifty percent of the hole drilled at the beginning of the experiment, 2 when over fifty percent of shell damage had been covered by newly produced shell, and 3 when repair was complete and the hole entirely covered by new shell (Figure 1b). Potential variation in the degree of shell repair among snails from different infection status could therefore be assessed over time. Both snails that died during the experiment and surviving individuals were used; degrees of shell repair 2–10 weeks postdrilling were estimated using snails that died during the experiment while shell repair at 12 weeks was assessed using surviving snails. Each snail was thus used only once in the dataset.

### 2.3 Snail survival, dissection. and measurements

Snails from each infection status were divided into eight groups of twelve or thirteen individuals, and each group was transferred to one of eight containers filled with 5 liters of natural seawater continuously aerated and maintained at 15°C. Sea lettuce (*Ulva* spp.) was provided ad libitum and ten empty shells of New Zealand clams *Austrovenus stutchburyi* were provided as grazing substrate and a source of calcium carbonate for shell production. Each one of the eight containers contained a comparable number of snails from each infection status (uninfected snails, *M*. *novaezealandense*,* Philophthalmus* sp., and double infections) and each shell treatment (undrilled, patched and drilled) for a total of 48–51 snails per container. Containers were cleaned, water replaced, and fresh sea lettuce added every 2 weeks for twelve weeks. Although pathogen concentration and diversity were not quantified, we used seawater collected from the same site as the snails and renewed regularly to maintain constant and natural pathogen levels. Snail survival was assessed every 2 weeks to test for potential effects of infection and shell damage on survival. Dead snails were recorded, removed, and preserved in 70% ethanol for later examination. At the end of the twelve‐week experiment, surviving snails were measured (total shell length in mm) and photographed to quantify shell growth as described above.

Surviving snails were then dissected to confirm their infection status and estimate caste‐ratio in snails carrying *Philophthalmus* sp. and double infections. The first, largest whorl of each snail shell was carefully cracked with a hammer, and the snail was removed from its shell as intact as possible (Leung & Poulin, 2011). The snail was transferred into a Petri dish filled with sea water and examined under a dissecting microscope. The visceral mass of the snail was then carefully teased apart to release parasite parthenitae and confirm snail infection status (Lloyd & Poulin, 2014a). When present, *Philophthalmus* sp. rediae were separated from snail tissue using fine tweezers. The number of reproductive (i.e., large, larvae‐producing morph) and nonreproductive rediae (i.e., small, soldier morph) was also counted and used to calculate a caste‐ratio (number of soldier rediae divided by the number of reproductive rediae) for each *Philophthalmus* sp. infected snail (Kamiya & Poulin, 2013; Lloyd & Poulin, 2013, 2014b). Note that snails that died during the experiment could not be used to estimate caste‐ratio in *Philophthalmus* sp. as both host and parasite tissues decompose very quickly after snail death, allowing confirmation of infection status but not accurate recording of the number of parasite parthenitae.

### 2.4 Statistical analyses

Snail size (i.e., shell length) ranged between 13.68 and 18.75 mm. However, snail sizes were evenly matched among the four infection status groups at the start of the experiment to standardize this variable across groups and eliminate any potential host‐size effects; mean shell length was 15.70, 15.60, 15.68, and 15.53 mm in uninfected, *Philophthalmus* sp., *M. novaezealandense* and double‐infected snails, respectively. Overall, there was no significant difference in snail size among infection statuses, shell treatments (undrilled, drilled and patched) or containers, and no significant interaction among any of these factors (ANOVA, 0.003 < *F*_2‐42,300_ < 1.274, .130 < *p *<* *.999).

Snail survival was analyzed using a Cox proportional hazard mixed effect model, using the function *coxme* in the package *coxme* (Therneau, 2015). This model provided a hazard response based on the time of death of individuals associated with a given treatment/infection status combination. Shell manipulation treatment (undrilled, patched, or drilled), infection status (uninfected, *Philophthalmus* sp. or *M. novaezealandense* single infected or double infection [*Philophthalmus* sp. and *M. novaezealandense*]), and time of death for individuals that died during the 12‐week period were recorded as fixed effects, in addition to censoring data to indicate snails that were alive at the end of the study, that is, right‐censored data. Container ID was included as a random effect to compensate for any unwanted variability caused by maintaining the snails in different containers.

Snail shell growth and caste‐ratio in *Philophthalmus* sp. were both analyzed using linear mixed effect models, using the *lmer* function in the package *lme4* in combination with the package *lmerTest* (Bates, Maechler, Bolker, & Walker, 2015; Kuznetsova, Brockhoff, & Christensen, 2016). In each model, fixed effects were infection status and shell treatment (undrilled or patched), with container ID again included as a random effect. Data from drilled snails were omitted from these analyses as very few drilled snails survived for the full 12‐week period (these data have, however, been included in the figure presented in the results to facilitate qualitative comparisons). Shell growth data were transformed using the function *powerTransform* in the package *car,* to meet the assumption of normality (Fox & Weisberg, 2011). Shell repair was also analyzed using a linear mixed effects model, although as the response variable was categorical (see description above), the model structure was slightly different and limited to individuals from the drilled and patched treatments. Fixed effects were infection status, shell treatment (drilled or patched), and time of death; note that in this case, “time of death” functions as the amount of time each snail had available to repair its shell (two to twelve weeks) rather than an indicator of survival. As with previously described models, container ID was included as a random effect. Where the effect of a factor was found to be significant (α = 0.05), *post hoc* pairwise comparisons were conducted between groups using the function *lsmeans* in the package *lsmeans* (Lenth, 2016). All analyses were completed using R v.3.2.3 (R Development Core Team 2015), and model diagnostics were checked for all linear mixed effect models.

## 3 RESULTS

**Figure 2:**
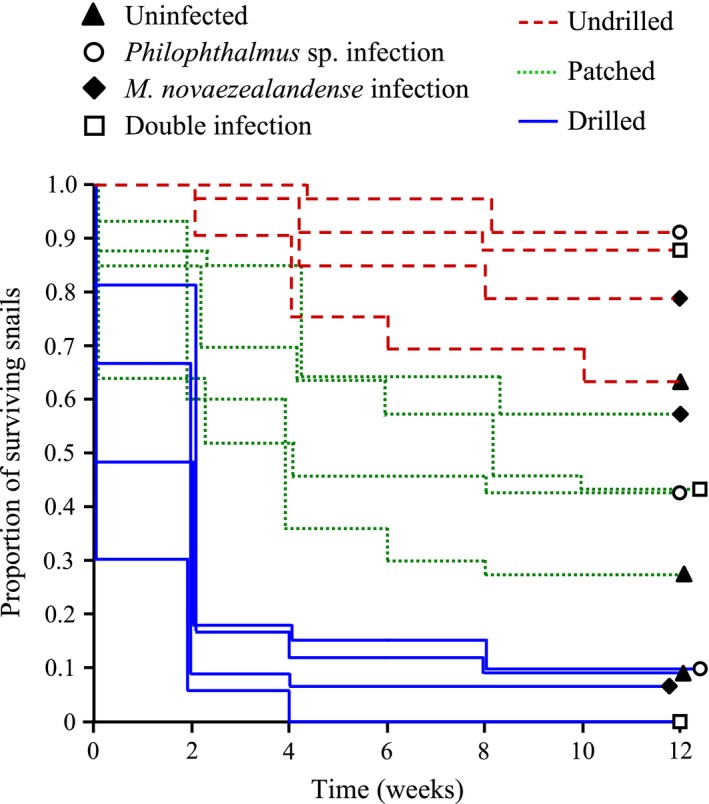
Kaplan–Meier plot showing snail survival (proportion of snails alive every 2 weeks) over the 12‐week period following shell manipulation (three treatments: undrilled, patched, or drilled) for uninfected individuals, snails harboring a single infection (*Philophthalmus* sp. or *Maritrema novaezealandense*), and individuals infected by both parasite species (double infection [*Philophthalmus* sp. and *M. novaezealandense*])

Survival of the snail *Z. subcarinatus* was significantly affected by shell manipulation (three treatments: undrilled, patched or drilled; χ² = 213.7, *p *<* *.0001) and by the interaction between shell manipulation treatment and snail infection status (four infection status uninfected, infected by either *M*. *novaezealandense* or *Philophthalmus* sp. [single infection], or both parasite species [double infection]; χ² = 26.7, *p *<* *.0001). However, infection status alone had no effect on snail host survival (χ² = 3.71, *p *=* *.293). Drilled snails exhibited the lowest survival (Figure 2); survival was significantly lower in drilled than undrilled (|*Z*| = 6.42, *p *<* *.0001) and patched snails (|*Z*| = 5.63, *p *<* *.0001), but not between undrilled and patched snails (|*Z*| = 1.88, *p *=* *.06) despite the trend observed in Figure 2.

**Figure 3:**
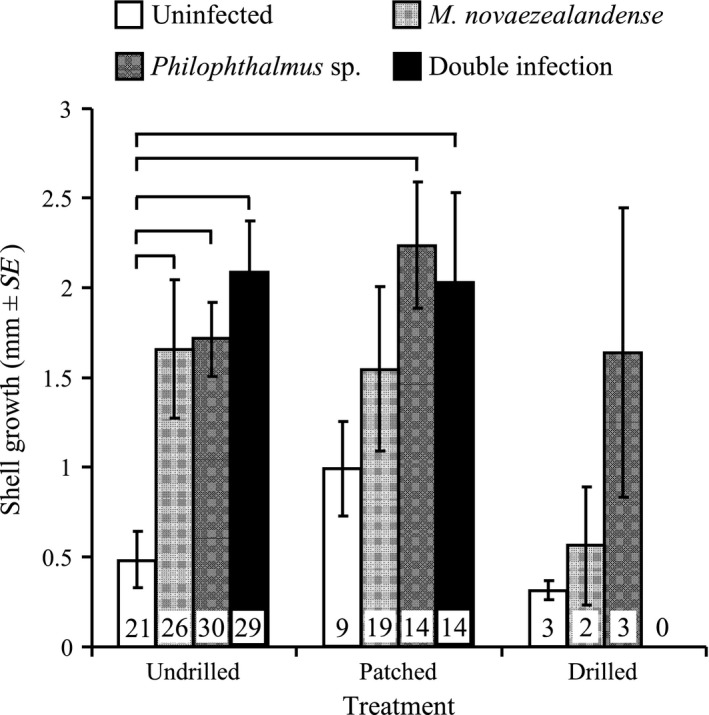
Mean shell growth (mm ± *SE*) shown by snails 12 weeks after shell manipulation (three treatments: undrilled, patched, or drilled) in uninfected snails, individuals harboring a single infection (*Philophthalmus* sp. or *Maritrema novaezealandense*) and snails infected by both parasite species (double infection [*Philophthalmus* sp. and *M. novaezealandense*]). Horizontal lines indicate significant differences between particular groups (α = 0.05). Numbers given inside bars are sample sizes; note that no drilled snail harboring a double infection survived the twelve‐week experiment

Shell growth was significantly affected by infection status only (*F*_1,145_ = 7.37, *p *<* *.0001). Generally, uninfected snails exhibited the lowest shell growth after 12 weeks (Figure 3; note that no snail harboring double infection survived the twelve‐week experiment in the drilled treatment). Shell growth in uninfected/undrilled snails was significantly lower than in snails from all other infection status in the undrilled treatment and that in snails harboring *Philophthalmus* sp. and double infections in the patched treatments (Figure 3). The shell manipulation treatment did not significantly affect shell growth in snails harboring trematode infections although drilled snails infected with *M*. *novaezealandense* displayed lower shell growth than undrilled and patched snails infected with the same parasite (Figure 3). The same pattern could be observed in uninfected snails, albeit not statistically significant (Figure 3). Shell growth in infected snails (*M*. *novaezealandense*,* Philophthalmus* sp., or double infection) was also generally higher than that in uninfected snails regardless of shell manipulation treatment (Figure 3). Note that high snail mortality and low sample size in some treatments and/or infection categories may have limited our ability to detect statistical significance among observed trends.

**Figure 4:**
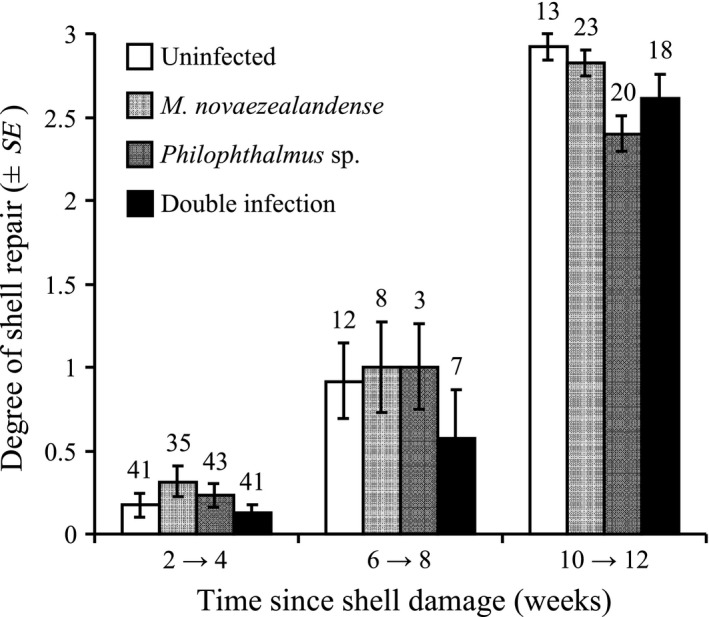
Degree of shell repair (mean ± *SE*) in uninfected snails, individuals harboring a single infection (*Philophthalmus* sp. or *Maritrema novaezealandense*) and snails infected by both parasite species (double infection [*Philophthalmus* sp. and *M. novaezealandense*]) shown over time since shell damage. Note that snails in the two and four, six and eight, and ten and twelve weeks postshell damage groups have been pooled in the figure, but not in the analyses presented in the text, to increase sample sizes and facilitate qualitative comparisons. Numbers above bars indicate sample sizes; drilled and patched snails are combined in the figure here as no difference in snail repair was found between these two treatments

Shell repair was significantly affected by infection status (*F*_3,248_ = 5.81, *p *<* *.001), time of death (*F*_1, 248_ = 426.67, *p *<* *.001), and the interaction of these two factors (*F*_3,248_ = 5.34, *p *=* *.001; Figure 4), but not by shell treatment (*F*_3,248_ = 2.48, *p *=* *.117). However, *post hoc* analyses failed to detect any significant differences among infection categories (Figure 4).

**Figure 5:**
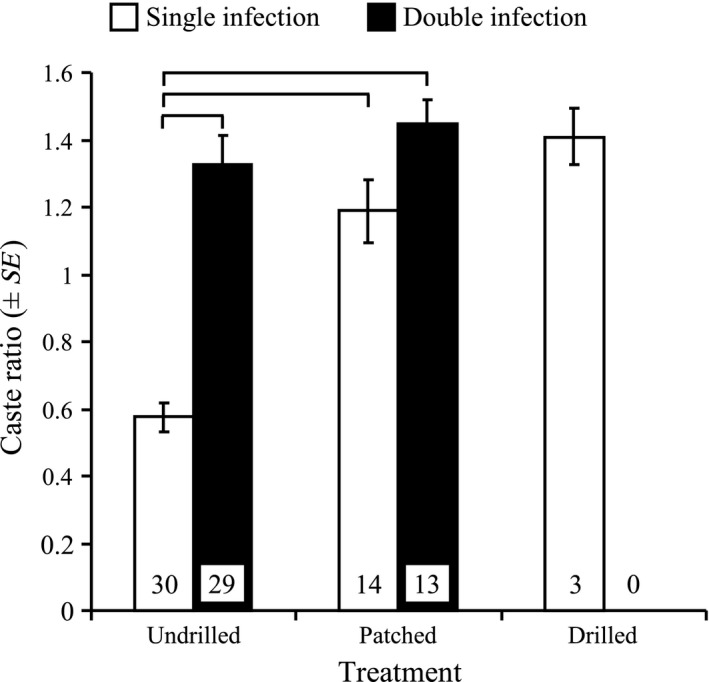
Mean caste‐ratio (±*SE*) in snails infected by *Philophthalmus* sp. 12 weeks after shell manipulation (three treatments: undrilled, patched or drilled) in host individuals harboring single (*Philophthalmus* sp. only) and double (*Philophthalmus* sp. and *Maritrema novaezealandense*) infections. Caste‐ratio is the number of soldier morph *Philophthalmus* sp. rediae divided by the number of reproductive morphs within an individual snail host. Horizontal lines indicate significant differences between particular groups (α = 0.05). Numbers given inside bars are sample sizes; note that no drilled snail harboring a double infection survived the twelve‐week experiment

Finally, caste‐ratio (i.e., proportion of rediae of each morph estimated as soldiers/reproductives) was significantly affected by treatment (shell manipulation; *F*_1,79_ = 20.7, *p *<* *.0001), infection status (*F*_1,76_ = 37.5, *p *<* *.0001), and the interaction of these two factors (*F*_1,76_ = 9.9, *p *=* *.002). Caste‐ratio in snails infected by *Philophthalmus* sp. only (single infection) was higher in patched and drilled snails than undrilled individuals although the difference was significant only between undrilled and patched treatments (Figure 5). While no snail host harboring double infections (*M*. *novaezealandense* and *Philophthalmus* sp.) survived the 12‐week experiment in the drilled treatment, individuals infected by both parasites exhibited higher *Philophthalmus* sp. caste‐ratios than snails with single infections in both patched and undrilled treatments (Figure 5). However, this difference was statistically significant in undrilled snails only (Figure 5).

## 4 DISCUSSION

Injuries are common in animal populations and generally expected to lead to decreased survival and reproduction. For example, shell damage is frequent in gastropods, ranging from 5% up to 90% of individuals depending on species and habitat (Andrews, 1935; Cadée, 1999; Cadée et al., 1997). Although quantitative data are scarce, injuries and subsequent shell repairs can have potentially costly consequences such as decreased survival and increased energetic requirements, potentially inducing a trade‐off in energy allocation between somatic growth or reproduction and shell repair (Geller, 1990). Although the histological mechanisms of shell regeneration in gastropods have been well studied, responses to injury and shell damage are often a neglected aspect of snail life‐histories. Marine snails are also frequently infected by trematode parasites known to influence key host life‐history traits such as survival, growth, and reproduction (Minchella, 1985; Sorensen & Minchella, 2001). Here, we found that shell damage, trematode infections, and the combination of both factors affected life‐history traits of the common mud snail *Z. subcarinatus*. Furthermore, we found that injury to the snail host can also have significant effects on a key life trait (i.e., caste‐ratio) of a trematode parasite with division of labor, *Philophthalmus* sp.

Snail survival was clearly affected by shell damage and its combination with trematode infections (Figure 2). As expected, damage to the shell significantly decreased snail survival (Day et al., 2000; Geller, 1990). However, and although a trend seemed apparent, snails that had the hole in their shell patched immediately after drilling did not display significantly increased mortality compared to undrilled individuals; only shell damage left exposed induced significant survival costs to *Z. subcarinatus* (Figure 2). Shell damage itself may actually have limited impact on snail survival and increased snail mortality following shell damage may instead be caused by subsequent secondary infections by pathogens (Andrews, 1935; Ducklow et al., 1979; Geller, 1990).

Trematode effects on snail host growth and survival can be highly variable, ranging from negative to positive depending on parasite virulence and life strategy or food availability to the snail host (Ballabeni, 1995; Minchella, 1985). Here, we found that infection status alone did not affect overall snail host survival. However, survival of the snail *Z. subcarinatus* was significantly affected by the interaction between shell manipulation treatment and infection status. Mortality rate in drilled snails was very high (>90%) regardless of infection status (Figure 2). In contrast, in both undrilled and patched treatments, survival of uninfected snails was significantly lower than that of trematode‐infected individuals. Higher mortality rates of uninfected *Z. subcarinatus* compared to infected conspecifics have been documented before and may be due to trade‐offs between survival and reproduction in uninfected snails; a trade‐off from which castrated infected snails are released (Fredensborg et al., 2005; Lloyd & Poulin, 2013). Parasites, including *Philophthalmus* sp., may also be able to alter the amount of resources taken from a host according to its stress and health levels to avoid over‐exploitation and parasite‐induced mortality (Jokela, Taskinen, Mutikainen, & Kopp, 2005; Lloyd & Poulin, 2013). Finally, there was no clear trematode species‐specific effects on snail host mortality despite the interspecific difference in parthenitae morphology and feeding strategies.

Because repairing shell damage is costly and energy allocated to shell regeneration cannot be used for reproduction or growth, snails are faced with a potential trade‐off between subsequent reproduction/growth and shell repair following damage (Kirkwood, 1981; Palmer, 1983, 1992). If shell regeneration detracts from reproduction and growth, repairs of damage to the shell should only occur if the damage induces decreased fitness (i.e., increased mortality or lower reproductive success). Here, it is clear that shell damage had strong negative effects on snail survival and should drive snails to invest in shell regeneration and rapid shell repair, regardless of their infection status. Accordingly, the degree and rapidity of shell repair were similar among snails from all infection statuses (Figure 4). We did not quantify potential decreases in reproductive output of snails during our experiments and cannot directly draw conclusions on a potential trade‐off between shell regeneration and reproductive success. However, trematode‐infected snails are completely castrated and de facto freed from this particular trade‐off. As a result, if there was a trade‐off between reproduction and shell regeneration, we would expect lower rates of shell regeneration in uninfected snails compared to trematode‐infected individuals. However, shell regeneration was similar in infected and uninfected snails, indicating a potential absence of trade‐off between reproduction and shell regeneration in *Z. subcarinatus* (Figure 4). However, as indicated previously, high mortality rates following shell damage may drive snails to invest heavily in shell regeneration regardless of its potential negative effects on reproduction.

Concomitantly with shell regeneration and damage repair, we quantified *Z. subcarinatus* growth rates among treatments. Shell growth is exclusively formed by the mantle skirt on the aperture's edge and only happens as the snail's body grows (Wagge, 1951). Shell growth is thus a direct indication of somatic growth in snails. Results indicate that shell treatment and shell regeneration had no deleterious effect on shell growth (Figure 3). This is not entirely surprising as shell regeneration following damage and normal shell growth result from two distinct histological and metabolic pathways in the snail (Geller, 1990; Watabe, 1983). However, both are energetically costly and the efficiency and rapidity of these mechanisms are highly dependent on resource availability (Geller, 1990; Wagge, 1951). Here, snails were fed ad libitum and abundant food resources likely allowed snails with drilled shells to increase energy intake and regenerate and grow new shell material concomitantly as indicated by the similar shell growth observed across all treatments (Figure 3).

Uninfected snails also exhibited significantly lower shell growth than trematode‐infected individuals in all shell treatments. This is consistent with previous studies on *Z. subcarinatus* and other gastropod species showing increased somatic and shell growth in infected snails compared to their uninfected counterparts (MacLeod & Poulin, 2015; Probst & Kube, 1999). Increased growth and larger body size in trematode‐infected snails is a frequent, although not universal, product of trematode infection induced by the energy made available by parasitic castration (Minchella, 1985; Mouritsen & Jensen, 1994; Probst & Kube, 1999). Energy freed up by parasitic castration is redirected into snail growth when more host resources than the parasite currently requires are made available (Sorensen & Minchella, 2001). Increased growth in infected snails is usually seen as a side‐effect of parasitic castration and was not affected by shell damage in *Z. subcarinatus* during our experiment (Mouritsen & Jensen, 1994; Probst & Kube, 1999). It has also been suggested that the degree to which snail growth is enhanced by trematode infection may depend on the type of parthenitae produced. Snails infected with rediae should show lower growth rates than snails infected with sporocysts as rediae feeding actively might cause more intensive damage to host tissues compared to sporocysts (Fernandez & Esch, 1991; Gorbushin, 1997; Sousa, 1983). However, we did not find such a pattern here indicating that *Philophthalmus* sp. rediae may induce little damage to snail tissues beyond castration.

There are costs and benefits to increasing the number of soldiers in a trematode colony with a division of labor. The colony should be better protected with increasing soldier numbers, but there could be an energetic cost to the production of soldiers (Miura, 2012). However, interspecific competition between *Philophthalmus* sp. and *M. novaezealandense* has fitness costs in terms of reproductive output for both species (Lloyd & Poulin, 2012). In the presence of competitors, the overall efficiency of a colony with division of labor should thus be enhanced by increased production of defensive caste members (Kamiya & Poulin, 2013; Mouritsen & Andersen, 2017). Accordingly, *Philophthalmus* sp. adjusts caste‐ratio in response to interspecific competition, increasing the proportion of nonreproductive soldiers (Lloyd & Poulin, 2013). Our results are consistent with previous findings; the proportion of soldiers was much higher in snails carrying double infections (Figure 5). Furthermore, our results indicate that the cost of shell damage and possible secondary infections is extremely high for snail hosts and therefore their parasites. Parasites with a division of labor should therefore invest heavily in the rapid production of nonreproductive soldiers to defend the colony, along with their host, against secondary microbial and fungal infections. As a result, in snail hosts infected with *Philophthalmus* sp. only, caste‐ratio in colonies from snails with shell damage was significantly higher than that in undrilled snails (Figure 5). However, while there was a slightly larger response in drilled versus patched snails, caste‐ratio was higher in *Philophthalmus* sp. only infected snails in both the drilled and patched snails compared to undrilled individuals, even though secondary microbial and fungal infections should be absent or very limited in patched individuals. Alternatively, caste‐ratio adjustment in snails with shell damage may thus simply be triggered by host trauma regardless of secondary pathogens, or actually initiated by shell damage in anticipation of potential secondary pathogen infections. This, however, cannot be inferred from our data, partly because survival of drilled snails was extremely low, partially impeding our ability to statistically test our hypotheses. Interestingly, snails carrying double infections did not show increased proportions of soldiers in *Philophthalmus* sp. colonies following shell damage. This may indicate that caste‐ratio in this parasite species, and potentially others with division of labor, can only vary within restricted values, responding nonspecifically to environmental fluctuations and subject to strong trade‐offs between defense and reproduction. Here, the short‐term costs and benefits of caste‐ratio adjustment *Philophthalmus* sp. colonies following shell damage to the host cannot be directly quantified. However, the similar response to interspecific competition and secondary infection risks by pathogens indicates that parasites with a division of labor benefit from the production of nonreproductive soldiers. As suggested before, nonreproductive rediae may actually play several, nonexclusive functional roles such as attacking competitor parasites and cleaning the colony of secondary pathogens (Mouritsen & Halvorsen, 2015). Finally, our results also highlight the general importance of ecological and environmental factors in influencing caste‐ratio in parasite colonies (Hechinger et al., 2011).

Overall, infections by trematode parasites did not seem to affect snail mortality, growth, or shell regeneration following shell damage. Snail survival was significantly affected by shell damage but trematode infections neither increased nor decreased the effects of such injury. However, our results clearly indicate that shell damage itself may not be the primary cause of increased mortality in *Z. subcarinatus*. Secondary infections by microbial or fungal pathogens may rather be responsible for the observed decreased snail survival as suggested previously in other species (Andrews, 1935; Ducklow et al., 1979; Geller, 1990; Morley, 2010). This is further indicated by the increase in the proportion of nonreproductive soldiers in *Philophthalmus* sp. colonies following shell damage in snail hosts. Caste‐ratio response to host shell damage was actually similar to that induced by interspecific competition with *M. novaezealandense*. *Philophthalmus* sp. colonies may thus be able to detect external threats beyond that of interspecific competition and respond accordingly by altering caste‐ratio to defend the colony and potentially its snail host. However, the rapidity of that response and the time‐lag between the signal of environmental fluctuations and caste‐ratio adjustment could not be determined here. Generally, division of labor has now been documented in several trematode species and provides models and opportunities to test for the universality of caste‐ratio responses to environmental fluctuations and for comparing ecology and evolution of social organization in animal societies (Kamiya & Poulin, 2013; Miura, 2012).

## CONFLICT OF INTEREST

None declared.

## AUTHORS' CONTRIBUTIONS

CM, CL, and RP conceived the ideas and designed methodology; RP acquired the funding; CL and RP completed the field collection of snails; CM and CL collected the data; CM analyzed the data; CL led the writing of the manuscript with significant contribution from CM and RP. All authors contributed critically to the drafts and gave final approval for publication.

## DATA ACCESSIBILITY

Data used in this manuscript will be made available in Dryad Digital Repository if accepted for publication in Functional Ecology.
